# Frontal bone tuberculosis presenting with blindness in a 14-year-old girl: a case report

**DOI:** 10.4076/1752-1947-3-8220

**Published:** 2009-06-17

**Authors:** Mohammad Shameem, Talha Saad, Rakesh Bhargava, Zuber Ahmad, Nazish Fatima, Haris Khan, Fakhrul Huda

**Affiliations:** 1 Department of Tuberculosis and Chest Diseases, Jawaherlal Nehru Medical College, Aligarh Muslim University, Aligarh U.P., 202002, India; 2 Department of Microbiology, Jawaherlal Nehru Medical College, Aligarh Muslim University, Aligarh U.P., 202002, India; 3 Department of Neurosurgery, Jawaherlal Nehru Medical College, Aligarh Muslim University, Aligarh U.P., 202002, India

## Abstract

**Introduction:**

The occurrence of tuberculosis in the flat bones of the skull is very rare. Only eight cases of tuberculosis of the frontal bone have been reported in the literature.

**Case presentation:**

A 14-year-old girl of Asian ethnicity presented with gradual loss of vision. A computed tomography scan of her head showed a diffuse, homogeneously ill-defined hyperdense lesion of size 2.9 × 5.3 cm (anteroposterior × thickness) involving the right orbit. Biopsy of the lesion confirmed the presence of epithelioid cells and Langerhans giant cells with caseous material. After surgical debridement with antitubercular treatment, the patient had an uneventful recovery.

**Conclusion:**

Although rare, tuberculosis can affect the flat bones of the skull. Tuberculosis of the frontal bone can be included in the differential diagnosis of blindness.

## Introduction

The occurrence of tuberculosis (TB) in the flat bones of the skull is very rare. With the global resurgence of tuberculosis, there have been reports of unusual sites being affected by the disease. Primary TB of the skull has been observed in the last century [1], but it is rare even in endemic areas. According to Chambers et al. [2], the rare occurrence of TB of the flat bones is due to its peculiar blood supply which does not allow tubercular bacilli to settle there. Skeletal TB accounts for 1% of all tuberculosis infections. Primary TB of the skull is very rare [3]. Only eight cases of tuberculosis of the frontal bone have been reported in the world literature. The youngest patient affected by tuberculosis osteitis of the frontal bone was a 3-year-old boy presenting with a 12-month history of sinuses over the frontal bone [4]. Frontal bone tuberculosis presenting with blindness is extremely rare, and not a single case has been reported so far in the medical literature.

## Case presentation

A 14-year-old girl of Asian ethnicity presented in the outpatient department of our hospital with loss of vision in her right eye for the previous 1.5 months, and a painless, discharging sinus over the front of her head on the right side for the preceding 14 months. The patient gave a history of low grade fever with intermittent discharge of cheesy material from the sinus. There was no history of chronic cough, dyspnea, loss of appetite or weight loss. The patient was malnourished (PEM grade-II). On examination, the sinus was found to be attached to the underlying frontal bone. The base of the sinus was non-tender. On pressing around the base, a few drops of cheesy material were extruded. Two posterior auricular lymph nodes were found enlarged on the right side and these were non-tender, and freely mobile. No other lymphadenopathy was detected. The chest and other systemic examinations were non-contributory.

**Figure 1:**
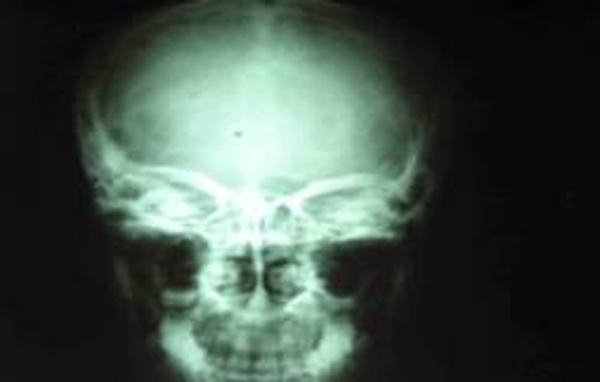
**X-ray of the skull showing a well defined area of radiodense shadow overlying the right frontal bone with sella appearing normal**.

**Figure 2:**
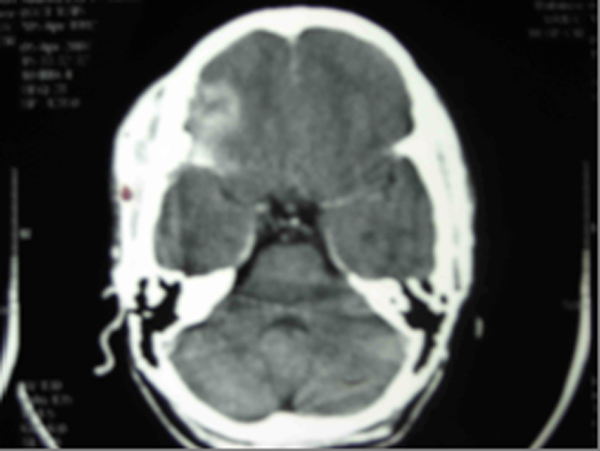
**A computed tomography scan of the head showing a diffuse, homogeneously ill defined hyperdense lesion of size 2**.9 × 5.3 cm (anteroposterior × thickness) involving the right orbit in its superior aspect with inferior and downward displacement of the right globe with extension into the anterior cranial fossa involving the right frontal region and the basal cistern region mainly on the right side.

**Figure 3:**
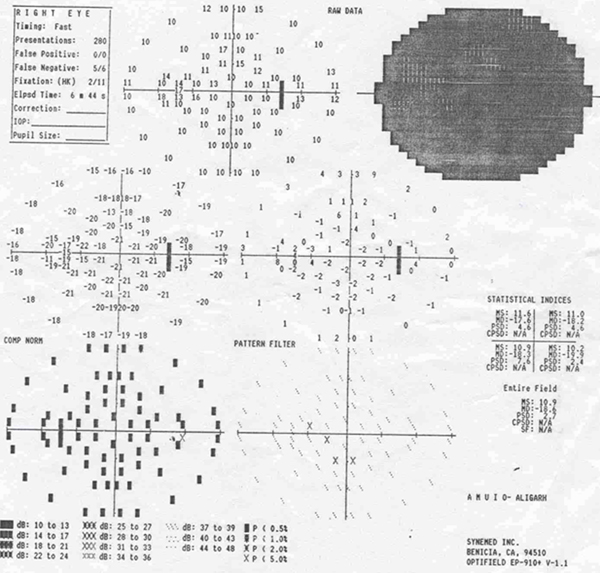
**Perimetry was also done and showed complete loss of vision on the right side with mean sensitivity (MS) 26**.8, mean deviation (MD) -18.6 and pattern standard deviation (PSD) 2.2.

The investigations revealed haemoglobin 10.5 gm%, erythrocyte sedimentation rate (ESR) 24 mm in the first hour (Wintrobe's Method), total lymphocyte count (TLC) 6200 cells/mm^3^ with polymorphs 76% and lymphocytes 24%. The Mantoux test was positive with induration of 16 mm × 18 mm. Polymerase chain reaction (PCR) was positive for tuberculosis, and direct smear was positive for acid fast bacillus (AFB). Chest X-ray was normal. X-ray of her skull (Figure 1), showed a well defined area of radiodense shadow overlying the right frontal bone with sella appearing normal. A computed tomography (CT) scan of her head (Figure 2) was done and showed a diffuse, homogeneously ill defined hyperdense lesion of size 2.9 × 5.3 cm (anteroposterior × thickness) involving the right orbit in its superior aspect with inferior and downward displacement of the right globe with extension into the anterior cranial fossa involving the right frontal region and the basal cistern region mainly on the right side; it also showed a mass effect with a midline shift toward the left. Lytic bone lesions involving the greater wing of the sphenoid, the roof of the medial wall of the orbit and the temporal bone on the right side with bony spikes were seen scattered in the right frontal region. Histopathology of tissue bone aggregates showed multiple granulomas composed of epithelioid cells, Langerhans giant cells and lymphocytes with casseous necrosis consistent with tuberculosis. Perimetry was also done and showed complete loss of vision on the right side with mean sensitivity (MS) 26.8, mean deviation (MD) -18.6 and pattern standard deviation (PSD) 2.2 (Figure 3).

A diagnosis was made of tuberculosis of the frontal bone with orbital extension. Surgery was performed 8 cm parallel to the hair line and behind it and the tumor infiltrating into the sphenoid, frontal and lateral wall of the orbit was removed. Antituberculosis treatment of directly observed therapy (DOTS) with rifampicin (10 mg/kg), pyrazinamide (25 mg/kg), ethambutol (15 mg/kg) and isoniazid (5 mg/kg) according to body weight was started. The four drugs were given for a period of 3 months followed by rifampicin (10 mg/kg) and isoniazid (5 mg/kg) for the next 9 months. The sinus healed in 3 weeks and radiological recovery was noted 4 months after the start of the antituberculosis treatment because of good compliance on the part of the patient. The patient's vision also improved as monitored by perimetry and refraction, and at the time of reporting, her vision was 6/60. Radiological recovery was in the form of sclerosis around a former lytic area.

## Discussion

The cause of blindness in this patient was probably due to extension of granuloma, which resolved after antitubercular treatment and the patient's vision improved gradually.

Because of its rarity, the diagnosis of tuberculosis of the skull bones has to be made after biopsy and histopathological confirmation to differentiate it from radiologically similar skull lesions, for example, eosinophilic granuloma. According to Zahorska et al. [5] who reported two cases of frontal bone tuberculosis, the frontal bone is rarely the site of tuberculosis. It was felt that disease at this unusual site occurs more commonly in patients on long-term corticosteroid therapy and they advised animal inoculation as the best diagnostic aid in clinically perplexing cases. Radiology is not diagnostic, and the diagnosis must be established by microbiological and histologicalstudies [6,7]. Although a definitive diagnosis requires biopsymaterial with granulomas and/or caseation complemented by acid-faststaining and culture, PCR detection of mycobacterial DNA in paraffin-embedded tissue has been used successfully in recent studies [8,9]. Tuberculous osteomyelitis of other cranial bones is also a rare entity. Hiranandani [10]reported a case of tuberculous petrositis in a 12-year-old child. The reason for the rare occurrence of calvarial tuberculosis even in endemic areas is not known. According to Chambers et al. [2], the rare occurrence of cranial and calvarial tuberculosis is due to the peculiar blood supply of flat bones which makes it difficult for *Mycobacterium tuberculosis* to settle there. The management of tuberculosis of flat bones of the skeleton is, by and large, conservative, with anti-tuberculosis therapy, rest to the area concerned and good nutrition.

In recent years, there has been an increase in surgical interventions so as to hasten healing. These range from simple debridement to 'excision of the focus' which involves extensive excision of all that is diseased. In our patient too, surgery was carried out and complete excision of the focus was performed. Surgical removal is required in patients with giant sequestra or where the response to conservative treatment of 4 to 6 weeks is not satisfactory.

DOTS therapy has revolutionized antitubercular treatment. The standardized DOTS system is used worldwide, and it is hoped that DOTS will be a major player in the global elimination of tuberculosis. Because DOTS is used globally, success of DOTS programs can be easily compared, allowing nations which have not adopted the program to see the potential for success.

## Conclusion

Although rare, tuberculosis can affect the flat bones of the skull. Tuberculosis of the frontal bone can be included in the differential diagnosis of blindness.

## Abbreviations

AFB: acid fast bacillus; CT: computed tomography; DOTS: directly observed therapy; ESR: erythrocyte sedimentation rate; PCR: polymerase chain reaction; TB: tuberculosis; TLC: total lymphocyte count.

## Consent

Written informed consent was obtained from the patient's father for publication of this case report and any accompanying images.

## Competing interests

The authors declare that they have no competing interests.

## Authors' contributions

MS diagnosed the patient as a case of tuberculosis, TS collected the requisite literature, RB and ZA carried out the printing work and cross-checked the article, HK and NF performed the Mantoux test, FH performed the surgery. All authors read and approved the final manuscript.
